# *Biomédica* en *PubMed Central* ^*®*^

**Published:** 2020-12-09

**Authors:** Luis Alberto Gómez-Grosso

**Affiliations:** 1 Editor, revista Biomédica; investigador, Grupo de Fisiología Molecular, Instituto Nacional de Salud; profesor catedrático, Departamento de Ciencias Fisiológicas, Facultad de Medicina, Universidad Nacional de Colombia; miembro correspondiente, Academia Colombiana de Ciencias Exactas Físicas y Naturales Universidad Nacional de Colombia Departamento de Ciencias Fisiológicas Facultad de Medicina Universidad Nacional de Colombia Colombia

La publicación de artículos en revistas científicas es el principal método para la comunicación del conocimiento científico validado por pares y es uno de los criterios fundamentales para evaluar la producción de investigadores, grupos de investigación e instituciones. Los editores de las revistas de salud y biomedicina deben privilegiar la calidad científica de los contenidos que se publican con la expectativa de su eventual impacto en la investigación, la educación, la práctica de las diferentes especialidades médicas y la adopción de decisiones.

En la era de la digitalización y el acceso abierto, una de las mejores herramientas para mejorar la visibilidad y el impacto de las publicaciones es la indexación y el registro de las revistas científicas en los más prestigiosos índices y repositorios internacionales que, además de velar por la calidad científica y editorial, son confiables y facilitan el acceso a los contenidos completos. En este contexto, nos complace informarles que el 10 de junio pasado, en m_e_dio de la pandemia causada por el SARS-CoV-2, recibimos la excelente noticia de la aceptación formal de *Biomédica* en *PubMed Central* ^*®*^ *,* lo que constituye un nuevo hito en su trayectoria.

Queremos en esta ocasión compartir con nuestros lectores esta buena nueva y explicar brevemente lo que significa que *Biomédica* hoy haga parte de ten prestigioso repositorio. ¿Qué es *PubMed Central* ^*®*^ *?* ¿Qué representa para la comunidad científica el hecho de que nuestra revista haya sido allí indexada? ¿Cuáles son algunos de los factores de éxito y qué podemos esperar en adelante?

*PubMed Central* ^*®*^ es el repositorio digital más prestigioso de artículos de revistas biomédicas y de ciencias de la vida. Fue creado por iniciativa del *National Center for Biotechnology Information* (NCBI), hace parte dela *National Library of Medicine* de los *Nation al Institutes of Health* de los Estados Unidos de América, y permite el acceso gratuito a los textos completos que reposan en sus bases de datos.

*PubMed Centíal* ^*®*^ se inició en el año 2000 con los artículos de dos revistas: *Proceedings of the National Academy of Science of the United States ofAmerica* y *Molecular Biology of the Cell.* Actualmente, contiene más de 6,5 millones de registros de textos completos de miles de revistas, que abarcan varios siglos de investigación biomédica y de las ciencias de la vida, desde finales de 1700 hasta la actualidad.

En el repositorio se almacenan y se cruzan las referencias de diversas fuentes en un formato común que les permite a los usuarios buscar y ubicar rápidamente todo el material relevante que se encuentre en la colección de artículos. La estructura del archivo de *PubMed Central* ^*®*^ también posibilita la integración de la literatura con otros recursos, por ejemplo, *PubMed Central* ^*®*^ internacional, el *GenBank,* las secuencias de referencia, el ómnibus de expresión genética, el visor de datos de genomas, el genoma humano y el del ratón, y diversos genomas virales, entre otras herramientas desarrolladas por sus creadores.

A partir de junio del 2020, *PubMed Central* ^*®*^ también incluye la publicación previa de los resultados de las investigaciones financiadas por los *National Institutes of Health.* Actualmente, después de las evaluaciones científicas y técnicas de rigor, más de 2.420 revistas han sido seleccionadas para su inclusión en *PubMed Central* ^*®*^ (https://www.ncbi.nlm.nih.gov/pmc/) ^1^.

La incorporación de una publicación en este repositorio significa que se la reconoce como parte de la literatura biomédica y de ciencias de la vida de la más alta calidad, y que su aporte contribuye al conocimiento científico como un patrimonio de la humanidad. El acceso abierto a la información científica en medios digitales es una de las herramientas que facilitan la rápida difusión de la información, el cual ha sido apoyado por *Biomédica,* y también, proporciona un espacio para que los científicos compartan las publicaciones y los datos así conservados, lo cual puede estimular la cooperación científica.

*Biomédica* está indexada en *Medline* desde el 2002 ^2^ y, a partir de junio de este año, los artículos depositados en *PubMed Central* ^*®*^ se pueden recuperar junto con los indexados en *Medline* mediante la plataforma *PubMed,* que es, posiblemente, el principal punto de búsqueda de la mayoría de los investigadores, profesionales y autores en el campo de la biomedicina. La funcionalidad de la plataforma *PubMed* y el acceso abierto a los textos completos en *PubMed Central* ^*®*^ permiten recomendarlo para las búsquedas sistemáticas de la literatura científica en este campo ^3^.

Este logro de la revista constituye un reconocimiento al trabajo en equipo, a las contribuciones de los autores, a la participación activa y comprometida de innumerables evaluadores, a la rigurosa revisión editorial y de crítica científica de los manuscritos, a la visión de sus editores, al apoyo continuo del Instituto Nacional de Salud de Colombia y, por supuesto, a una incansable labor logística y de asistencia editorial a cargo de un excelente grupo de apoyo.

La inclusión en *PubMed Central* ^*®*^ es señal de reconocimiento académico, un logro que exige esfuerzos para seguir cumpliendo con las obligaciones aceptadas, es decir, los estándares científicos, éticos y editoriales exigidos por la *National Library of Medicine* y los *National Institutes of Health.* En este sentido, se deben revisar y actualizar periódicamente las instrucciones para los autores, las consideraciones éticas de la publicación, la revisión por pares, los conflictos de intereses, los derechos de autor, la licencia de distribución y la asignación de los identificadores de objetos digitales *(digital object identifbr,* doi); en pocas palabras, cumplir y hacer cumplir las recomendaciones editoriales que periódicamente emiten el *International Committee of Medical Journal Editors* y el *Council of Science Editors* para mantener un buen estándar científico y editorial. Además, también ayudan el uso del formato *Journal Article Tag Suite* (JATS) y del *eXtensible Markup Language* (XML), así como otros ajustes esenciales para garantizar el óptimo acceso abierto ^4^^,^^5^.

Otros requisitos incluyen la publicación de artículos en inglés bien editados, la validación de las referencias de los artículos y el uso de referencias cruzadas, también útiles para la navegación en la plataforma de *PubMed Central* ^*®*^ y la consulta de los artículos indexados en *Medline.* La incorporación en *PubMed Central* ^*®*^ amplía las posibilidades de visibilidad de un artículo y de su eventual citación, porque las plataformas de *PubMed* y *PubMed Central* ^*®*^ están equipadas con complementos para la promoción posterior de la publicación por medio de las redes sociales.

Esperamos que la inclusión de *Biomédica* en *PubMed Central* ^*®*^ nos ayude a seguir aumentando la visibilidad de la revista y, en consecuencia, la posibilidad de generar un mayor impacto en la comunidad científica nacional e internacional y en la sociedad en general, especialmente entre las comunidades de habla hispana. Aspiramos, asimismo, a mantenernos sintonizados con las tendencias editoriales globales a la par con los numerosos medios de publicación en Medicina Tropical y Salud Pública, para servir mejor a las comunidades científicas y ampliar nuestra influencia social y la promoción posterior de los contenidos publicados, lo que, muy seguramente, se verá facilitado por nuestra inclusión en *PubMed Central* ^*®*^ *.*

Una vez más, queremos reiterar nuestros agradecimientos a *PubMed Central* ^*®*^ por haber evaluado nuestra revista y por haberla aceptado en tan importante repositorio científico. Asimismo, debo resaltar nuevamente que este logro es el resultado del trabajo de los muchos editores que han formado parte del Comité Editorial, así como del creciente número de revisores expertos, nacionales e internacionales, y necesariamente del número creciente de autores colombianos y latinoamericanos que han confiado en la calidad científica y editorial de la revista. A todos ellos les agradecemos sus esfuerzos y por habernos seleccionado como la tribuna para la publicación de sus manuscritos.

Por último, queremos manifestar públicamente nuestro especial agradecimiento a todo el equipo editorial de *Biomédica* -Comité Editorial y Comité Científico- y, especialmente al grupo de apoyo editorial y administrativo de la revista -asistente editorial, correctores de estilo, diseño y diagramación, y mercadeo digital- y a las directivas del Instituto Nacional de Salud por su irrestricto apoyo al sostenimiento y desarrollo de la revista. Esperamos que toda la comunidad científica nacional e internacional continúe brindando el apoyo necesario para poder mantener la calidad y el rigor científico y editorial que han caracterizado a la revista *Biomédica* durante los últimos cuarenta años.
